# Adoptive transfer of immune subsets prior to MCAO does not exacerbate stroke outcome in splenectomized mice

**DOI:** 10.15761/jsin.1000105

**Published:** 2015-09-28

**Authors:** Jianming Wang, Abby L. Dotson, Stephanie J. Murphy, Halina Offner, Julie A. Saugstad

**Affiliations:** 1 Department of Anesthesiology and Perioperative Medicine, Oregon Health and Science University, Portland, OR, USA; 2 Department of Neurology, Oregon Health and Science University, Portland, OR, USA; 3 Neuroimmunology Research, VA Medical Center, Portland, OR, USA; 4 Department of Behavioral Neuroscience, Oregon Health and Science University, Portland, OR, USA; 5 Department of Medical and Molecular Genetics, Oregon Health and Science University, Portland, OR, USA

**Keywords:** inflammation, focal cerebral ischemia, middle cerebral artery occlusion, experimental stroke, CD4, CD8, CD11b, splenectomy, female mice, immune modulation

## Abstract

The peripheral immune response contributes to neurologic impairment after stroke and the extent of initial damage is greater in males than females. We have previously shown that spleen cells directly contribute to ischemic damage in males, as splenectomy prior to experimental stroke eliminates the sex differences in infarct volume. This study aims to determine which specific subset of immune cells exert pathogenic effects when injected 24 hours before MCAO induction into splenectomized male and female WT mice. The results demonstrate that CD4/CD8/CD11b treated mice had no significant effect on infarct volumes *vs*. vehicle-treated control mice after MCAO. However, there were significant alterations to the resident peripheral immune composition. These results suggest that there are regulatory factors resulting from splenectomy or other possible influences that inhibit peripheral immune cell contribution to neuroinflammation and thus contributing to differential effects of the spleen on stroke outcome in males and female mice.

## Introduction

The 2015 Heart Disease and Stroke Statistics Update (which is compiled annually by the American Heart Association, the Centers for Disease Control and Prevention, the National Institutes of Health and other government sources) lists stroke as the number four cause of death and the leading cause of disability in the United States [1]. Stroke is also the second-leading global cause of death behind heart disease, accounting for 11.13% of total deaths worldwide [1]. We, and others, have examined the role of the immune response in the pathogenesis of ischemic stroke. Following stroke, activation of leukocytes in the spleen causes release of immune cells into the blood, infiltration of leukocytes and activation of microglial cells in the brain, and a resultant increase in infarct volume [2–5]. There are reported differences in the spleen between male and female mice following MCAO. Male rats that undergo splenectomy prior to experimental stroke show reduced infarct volume and neurological impairment due to a decrease in T cells, neutrophils, macrophages and pro-inflammatory cytokines, and an increase in anti-inflammatory cytokines, in brain tissue [6]. Irradiation of the spleen 4 hours after MCAO in male mice also prevented deployment of splenocytes resulting in decreasing microglia and infiltrating T cells in the ischemic brain and reduced infarct volume [7]. However, the effect of the spleen on infarct volume and stroke progression was relatively unknown in female animals, thus we recently examined the effect of splenectomy on responses to focal cerebral ischemia in female mice. Splenectomy or sham splenectomy was performed in male and female mice two weeks prior to 60 min duration of transient middle cerebral artery occlusion (MCAO). We then examined infarct volume and peripheral and ischemic brain specific immunological parameters following 96 hours of reperfusion, and found that sex differences in infarct volume after stroke were abolished by splenectomy [8]. Further, activated T cells in the periphery correlated with the sex and splenectomy differences in infarct volume, and CD11b^+^ monocytes were indicative of stroke outcome in male mice. The primary purpose of the present study was to determine the effect of adoptive transfer of CD4/CD8 T cells or CD11b monocytes/macrophages 24 h before the induction of MCAO on infarct volume in male and female splenectomized mice. Our results demonstrate that treatment with either quiescent or activated CD4/CD8 T cells or activated CD11b^+^ cells does not significantly increase infarct volume when administered 24 h prior to the onset of stroke in splenectomized mice. Further, these studies support that transfer of CD8^+^ T cells prior to MCAO leads to more potentially harmful immune cell subsets in the blood.

## Materials and methods

### Ethics statement

The study was conducted in accordance with National Institutes of Health guidelines for the use of experimental animals. The Institutional Animal Care and Use Committees at Oregon Health and Science University and the Portland Veteran Affairs Medical Center approved all protocols.

### Animals

Male and female C57BL/6J (wild-type, WT) mice weighing 20 to 25 g and 8 to 13 weeks of age (Jackson Laboratory, Sacramento, CA, USA) were used as recipients for adoptive transfers and induction of transient focal cerebral ischemia. All WT mice were housed at the Oregon Health and Science University. Male and female GFP mice (on a C57BL/6J background) were used at 8 to 10 weeks of age as donors for adoptive transfers. These mice were bred and housed in the Animal Resource Facility at the Portland Veterans Affairs Medical Center in accordance with institutional guidelines.

### Splenectomy

Splenectomy was done under isoflurane anesthesia (induction 5.0% and maintenance 3.0% delivered *via* a face mask in O_2_-enriched air). A longitudinal incision (10–15 mm) was made on the left dorsolateral side of the abdomen, caudal to the last rib, and then a 10 mm incision in the peritoneal wall was made. The splenic arteries and efferent venous were ligated with sterilized 6-0 silk sutures separately by looping the sutures through the mesentery. The mesentery and connective tissue were cut and the spleen removed. Abdominal muscle incisions and skin were separately closed using sterilized 6-0 absorbable sutures. Spleens were removed 14 days before MCAO.

### Leukocyte isolation from donor spleens

Spleens from individual GFP mice were removed and a single-cell suspension was prepared by passing the tissue through a 100 μm nylon mesh (BD Falcon, Bedford, MA). The cells were washed using RPMI 1640 and the red blood cells lysed using 1× red blood cell lysis buffer (eBioscience, Inc., San Diego, CA) and incubated for 1 min. The cells were washed with RPMI 1640, counted on a Cellometer Auto T4 cell counter (Nexcelom, Lawrence, MA), and resuspended in separation buffer (phosphate-buffered saline, pH 7.2, 0.5% bovine serum albumin, and 2 mM EDTA) for cell sorting.

### Cell sorting and adoptive transfer of T cells

Male and female GFP mice served as donors of T cells and monocytes/macrophages. Splenic CD4 or CD8 T cells were purified using paramagnetic bead-conjugated antibodies (Abs) from the CD4 or CD8 cell isolation kit, respectively, and subsequently separated by AutoMACS (MiltenyiBiotec, Auburn, CA). CD11b monocytes/ macrophages were purified using the Easysep negative selection CD11b kit (Stemcell Technologies, Vancouver, BC, Canada).

The negative fraction of the cells thus separated was CD4^+^, CD8^+^ or CD11b^+^ cells with a purity of ≥ 75%. Cells were suspended in sterile saline and counted using a hemocytometer with the trypan blue exclusion method. Twelve million purified CD4^+^, eight million CD8^+^ T cells or five million CD11b^+^ cells from the donor mice were suspended in 100 μL saline and were transferred intravenously (i.v.) *via* tail vein injection into splenectomized recipient WT mice (experimental group) 24 h before MCAO while the vehicle control group received 100 μL sterile saline.

### *In vivo* activation of immune cells with MOG

Male and female GFP mice between 8 and 12 weeks of age were immunized subcutaneously at four sites on the flanks with 0.2 mL of an emulsion of 200 μg myelin/oligodendrocyte glycoprotein (MOG) immunogenic peptide and complete Freund’s adjuvant containing 400 μg of heat-killed *Mycobacterium tuberculosis* H37RA (Difco, Detroit, MI, USA). Spleens were harvested eight days after immunization and processed as described for transfer.

### Transient focal cerebral ischemia

Transient focal cerebral ischemia was induced in male WT mice for 1 h by reversible right MCAO under isoflurane anesthesia followed by 96 h of reperfusion as previously described [9]. The individual performing all MCAO surgeries was blinded to treatment group. Head and body temperature were controlled at 36.0 ± 1.0°C during surgery, MCAO, and early reperfusion with warm water pads and a heating lamp. Occlusion and reperfusion were verified in each mouse by laser Doppler flowmetry (LDF) (Model DRT4, Moor Instruments, Inc., Wilmington, DE, USA). Occlusion was achieved by introducing a 6-0 nylon monofilament (ETHICON, Inc., Somerville, NJ, USA) with a silicone-coated (Xantopren comfort light, Heraeus, Germany) tip through an external carotid artery stump distal to the internal carotid artery to the origin of the middle cerebral artery. Adequacy of MCAO was confirmed by monitoring cortical blood flow at the onset of the occlusion with a LDF probe affixed to the skull. After occlusion was initiated, the incision was closed with 6-0 surgical sutures (ETHICON, Inc., Somerville, NJ, USA). At the end of the 1 h ischemic period, the occluding filament was withdrawn for reperfusion, and the skin incision was bonded with tissue adhesive (3M Vetbond, St. Paul, MN). Each mouse was then awakened and recovered in a separate cage with a warm water pad. Mice were survived for 96 hours following initiation of reperfusion.

### Neurological deficit scores

Neurological deficit scores were determined at baseline, after 5 minutes of reperfusion, then at 1, 2, 3, and 4 days post-occlusion (POD) to confirm ischemia and the presence of ischemic injury. We used a 0 to 5 point scale as follows: 0, no neurological dysfunction; 1, failure to extend left forelimb fully when lifted by tail; 2, circling to the contralateral side; 3, falling to the left; 4, no spontaneous movement or in a comatose state; and 5, death [10]. Any animal without a deficit at POD1 was excluded from the study.

### Cerebral blood flow analysis

The temporal LDF window was located in the middle between the outer canthus of right eye and right external auditory canal. A 5 mm skin incision was made, then tweezers and electric bipolar coagulation made a small hole in the temporal muscle. The pens of the bipolar coagulation do not touch the skull as far as possible, and power control of bipolar coagulation is 3. After making the temporal LDF window, the mouse was gently turned upside down in the supine position. The tip of metallic LDF probe was gently attached on the surface of skull and the laser Doppler probe was affixed on the bench to verify vascular occlusion and monitor cortical ischemia/reperfusion. The baseline value of LDF for MCAO procedures was over 300.

### Infarct volume analysis

Individual performing infarct volume analysis was blinded to treatment group. Mice were euthanized and brains collected at 96 h of reperfusion for 2,3,5-triphenyltetrazolium chloride histology and then digital image analysis of infarct volume was performed as previously described [10]. Images were analyzed using Sigma Scan Pro 5.0 Software (Systat, Inc., Point Richmond, CA). To control for edema, regional infarct volume (cortex, striatum, and hemisphere) was determined by subtraction of the ipsilateral non-infarcted regional volume from the contralateral regional volume. This value was then divided by the contralateral regional volume and multiplied by 100 to yield regional infarct volume as a percent of the contralateral region.

### Leukocyte isolation from blood

Blood was obtained from cardiac puncture 96 hours after MCAO. Red blood cells were lysed using 1× red blood cell lysis buffer (eBioscience, Inc., San Diego, CA) for 5 min at room temperature. The cells were washed with RPMI 1640, counted on a hemocytometer and resuspended in staining medium (PBS containing 0.1% NaN_3_ and 1% bovine serum albumin (Sigma, Illinois)) for flow cytometry.

### Analysis of cell populations by flow cytometry

All antibodies were purchased (BD Biosciences, San Jose, CA or eBioscience, Inc., San Diego, CA) as published. Four-color (FITC, PE, APC and PerCP) fluorescence flow cytometry analyses were performed to determine the phenotypes of splenocytes. One million cells were washed with staining medium, blocked with anti-mouse CD16/CD32 Mouse BD Fc Block™ (BD Biosciences, San Jose) for 15 minutes at 4°C and then incubated with combinations of the following monoclonal antibodies: CD4 (GK1.5), CD8 (53–6.7), CD11b (MAC-1), CD44 (IM7) and CD19 (ID3) for 20 min at 4°C. Data were collected with BD AccuriTM C6 software on a BD AccuriTM C6 (BD Biosciences, San Jose, CA).

### Statistical analysis

Infarct volume data are presented as mean ± SEM. Differences in regional infarct volumes were determined with one-way ANOVA. Functional outcomes for neurological deficit scores were analyzed by Mann Whitney Rank Sum test. Differences in peripheral immune frequencies were determined with Student’s t-test. Statistical significance was *p*<0.05. Statistical analyses were performed using GraphPad Prism version 6.0 (GraphPad Software, La Jolla, CA).

## Results

### Mortality and exclusions

Overall number of mice used for all of these studies combined was 143. Mortality for the CD4/CD8 adoptive transfer MCAO studies was 28 mice out of a total of 98 mice, with mortality ranging from 2 to 8 mice within the experimental groups. Mortality for the activated CD4/ CD8 adoptive transfer MCAO studies was 7 mice out of a total of 25 mice, with mortality ranging from 1 to 3 mice within the experimental groups. Mortality for the CD11b adoptive transfer MCAO studies was 2 mice out of a total of 20 mice, with mortality ranging from 0 to 1 mice within the experimental groups; 2 mice were excluded based on cerebral blood flow >30%. Causes of death included infection following splenectomy, subarachnoid hemorrhage during surgery, or failure of the adoptive transfer method.

### Adoptive transfer of CD4^+^ or CD8^+^ T cells to splenectomized male or female mice does not exacerbate infarct volume induced by focal ischemia

**Figure 1:**
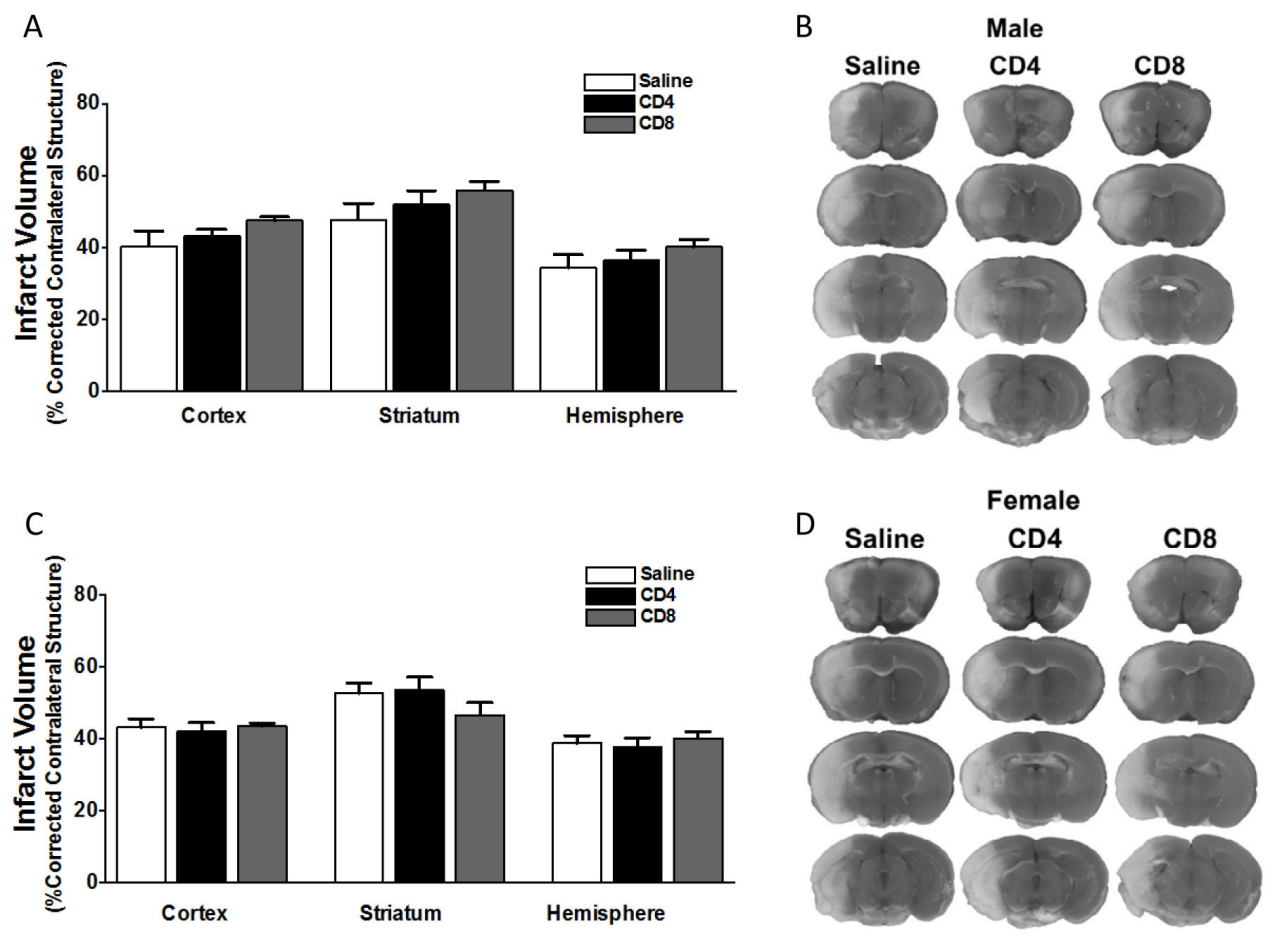
Adoptive transfer of CD4^+^ or CD8^+^ T cells to splenectomized male or female mice does not exacerbate infarct volume induced by focal ischemia Male and female mice were subject to splenectomy two weeks prior to transient MCAO (60 min). Mice were injected with CD4^+^ or CD8^+^ T cells 24 hours prior to MCAO. Brains were harvested 96 h after MCAO. (A) Infarct volumes measured as percentage of corrected contralateral structure for male mice injected with saline vehicle (n=10), 12 million CD4^+^ T cells (n=12), or 8 million CD8^+^ T cells (n=14) by intravenous transfer 24 h before MCAO. (B) Representative brain slices for male mice stained with TTC. Values represent mean numbers (± SEM) of splenectomized males per group analyzed using a one-way ANOVA. (C) Infarct volumes measured as percentage of corrected contralateral structure for female mice injected with saline vehicle (n=12), 12 million CD4^+^ T cells (n=11), or 8 million CD8^+^ T cells (n=11) by intravenous transfer 24 h before MCAO. (B) Representative brain slices for female mice stained with TTC. Values represent mean numbers (± SEM) of splenectomized females per group analyzed using a one-way ANOVA.

We examined the effect of adoptive transfer of CD4^+^ and CD8^+^ T cells on infarct volume in splenectomized mice. Male and female C57BL/6J WT mice underwent splenectomy two weeks prior to focal cerebral ischemia induced by MCAO. 24 h before MCAO the splenectomized mice were intravenously given saline (no cells), or a spleen equivalent number of CD4^+^ T cells or CD8^+^ T cells (12x10^6^ and 8x10^6^, respectively). The mice were subject to 60 min MCAO, followed by 96 h reperfusion when brains were evaluated for infarct volume. The results show that adoptive transfer of 12 million CD4^+^ T cells (n = 12), or 8 million CD8^+^ T cells (n = 14) had no effect on infarct volume in male mice, relative to injection of saline (n = 10) (Figure 1A). Representative TTC stains of cerebral sections of male mice 96 h after MCAO show that localization of the ischemic lesion did not differ between splenectomized male mice receiving intravenous saline (Figure 1B, left column) *vs.* CD4^+^ or CD8^+^ T cells (Figure 1B, right columns) 24 h before MCAO. Similarly, the results show that adoptive transfer of 12 million CD4^+^ T cells (n = 12), or 8 million CD8^+^ T cells (n = 11) had no effect on infarct volume in female mice, relative to injection of saline (n = 11) (Figure 1C). Representative TTC stains of cerebral sections of female mice 96 h after MCAO show that localization of the ischemic lesion did not differ between splenectomized male mice receiving intravenous saline (Figure 1D, left column) *vs.* CD4^+^ or CD8^+^ T cells (Figure 1D, right columns) 24 h before MCAO. All values represent mean numbers (± SEM) of splenectomized mice per group analyzed using a one-way ANOVA.

### Adoptive transfer of CD4^+^ or CD8^+^ T cells to splenectomized male or female mice has no effect on neurological deficit scores

**Figure 2:**
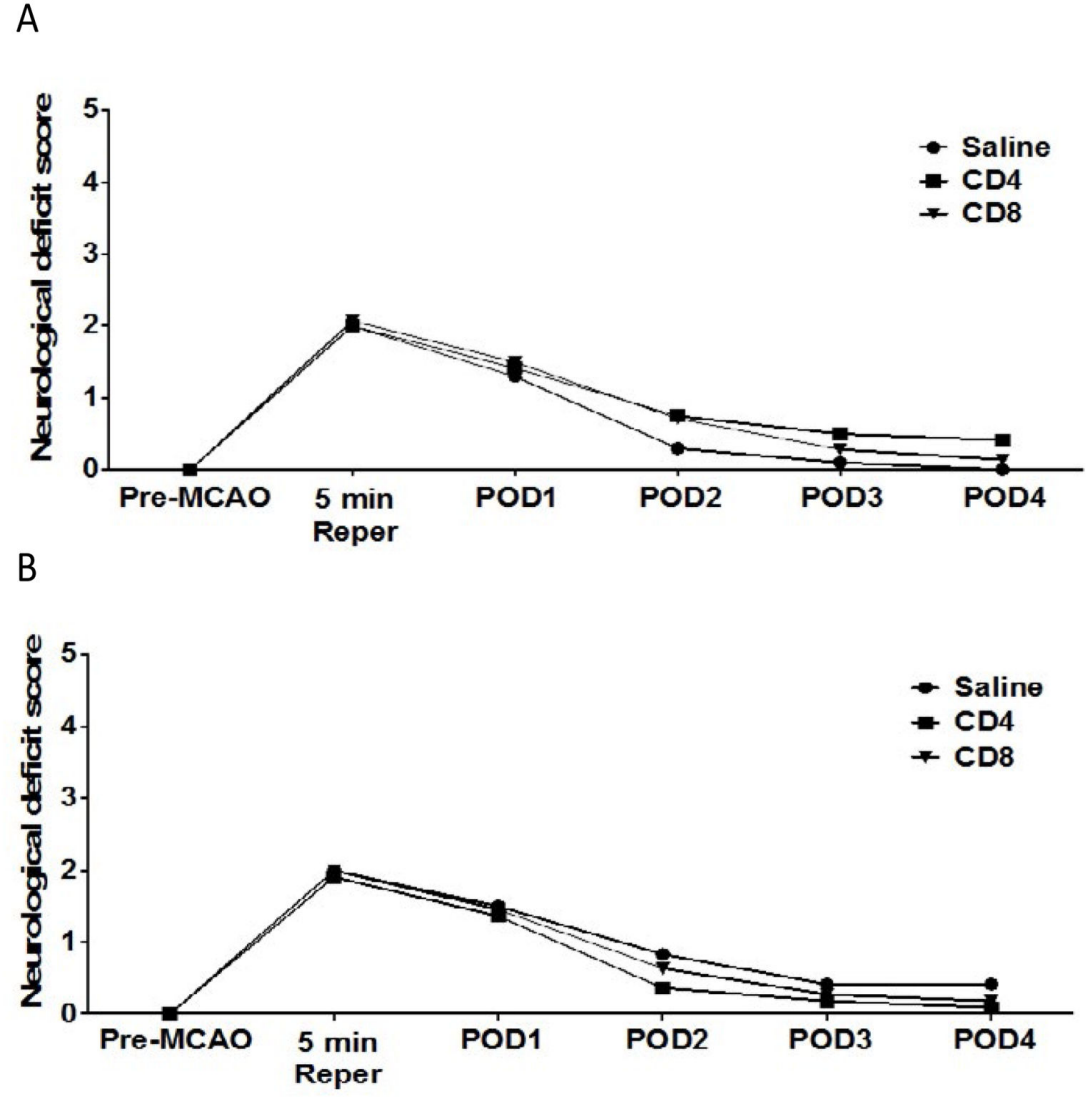
No effect of adoptive transfer of CD4/CD8 T cells on neurological deficit score in splenectomized male and female mice Male and female mice were subject to splenectomy two weeks prior to transient MCAO (60 min). Mice were injected with CD4^+^ or CD8^+^ T cells 24 hours prior to MCAO. Brains were harvested 96 h after MCAO. (A) Neurological Deficit Score measured in male mice injected with saline vehicle (n=10), 12 million CD4^+^ T cells (n=12), or 8 million CD8^+^ T cells (n=14) by intravenous transfer 24 h before MCAO. (B) Neurological Deficit Scoremeasured in female mice injected with saline vehicle (n=12), 12 million CD4^+^ T cells (n=11), or 8 million CD8^+^ T cells (n=11) by intravenous transfer 24 h before MCAO. Functional outcomes for neurological deficit scores were analyzed by Mann Whitney Rank Sum test. Statistical significance was p<0.05.

We examined the effect of CD4^+^ or CD8^+^ T cell injection 24 hours prior to MCAO in male and female splenectomized mice on neurological deficit scores. Figure 2 shows neurological deficit scores measured for male (A) or female (B) mice before MCAO (Pre-MCAO), after 5 minutes of reperfusion (5 min Reper), or 1, 2, 3 or 4 days post-occlusion (POD). The results show there is no effect of injection with saline vehicle (n = 10), 12 million CD4^+^ T cells (n = 12), or 8 million CD8^+^ T cells (n = 14) by intravenous transfer 24 h before MCAO. Functional outcomes for neurological deficit scores were analyzed by Mann Whitney Rank Sum test. Statistical significance was *p*<0.05.

### Adoptive transfer of CD4^+^ or CD8^+^ T cells to splenectomized male or female mice has no effect on cerebral blood flow

**Figure 3:**
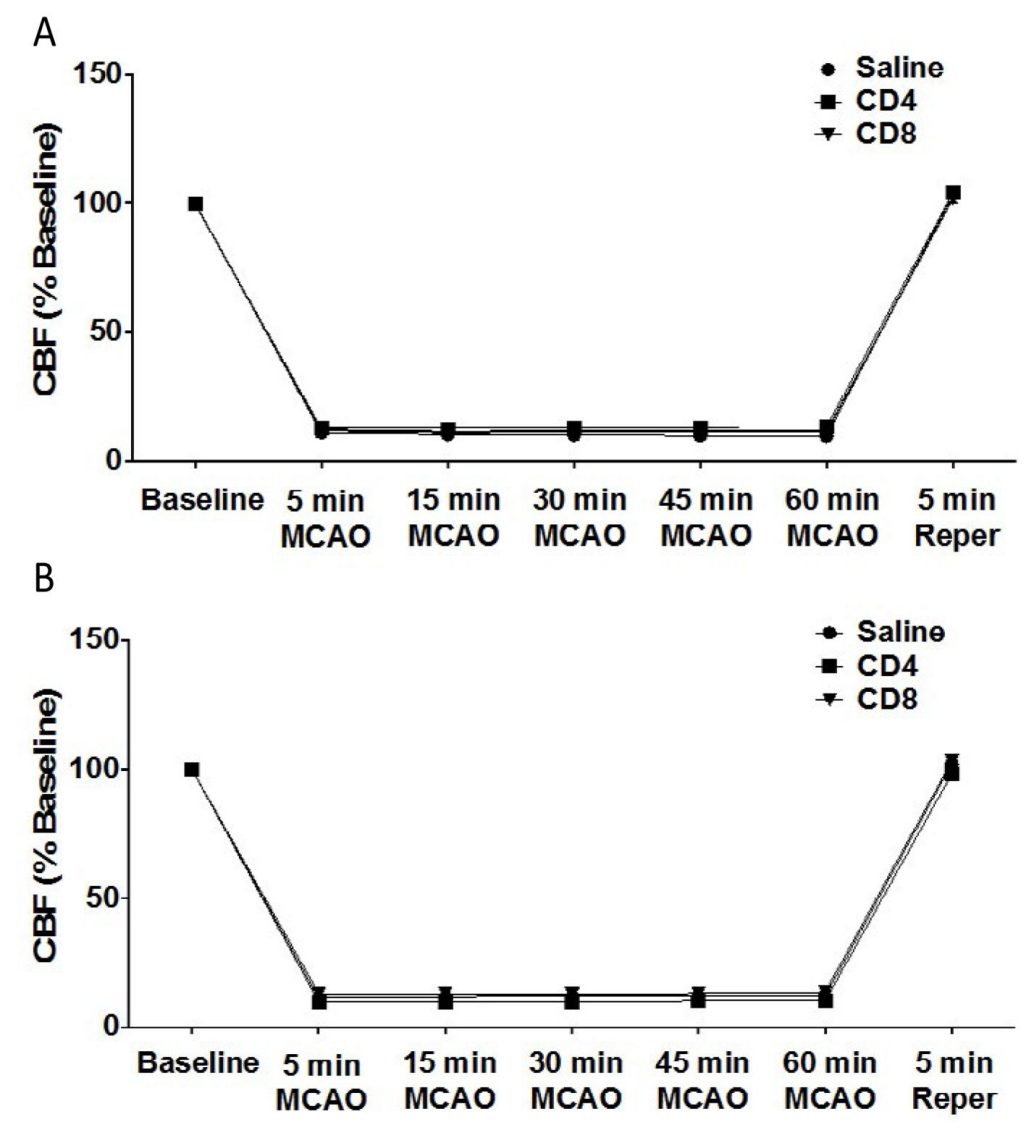
No effect of adoptive transfer of CD4/CD8 T cells oncerebral blood flow in splenectomized male and female mice Male and female mice were subject to splenectomy two weeks prior to transient MCAO (60 min). Mice were injected with CD4^+^ or CD8^+^ T cells 24 hours prior to MCAO. Brains were harvested 96 h after MCAO. (A) Cerebral blood flow measured in male mice injected with saline vehicle (n=10), 12 million CD4^+^ T cells (n=12), or 8 million CD8^+^ T cells (n=14) by intravenous transfer 24 h before MCAO. (B) Cerebral blood flow measured in female mice injected with saline vehicle (n=12), 12 million CD4^+^ T cells (n=11), or 8 million CD8^+^ T cells (n=11) by intravenous transfer 24 h before MCAO.

We examined the effect of CD4^+^ or CD8^+^ T cell injection 24 hours prior to MCAO in male and female splenectomized mice on cerebral blood flow. Figure 3 shows cerebral blood flow measures for male or female mice before MCAO (baseline), throughout MCAO surgery (5, 15, 30, 45, and 60 min MCAO), and after 5 minutes of reperfusion (5 min Reper). The results show there is no effect of injection with saline vehicle (n = 10), 12 million CD4^+^ T cells (n = 12), or 8 million CD8^+^ T cells (n = 14) by intravenous transfer 24 h before MCAO on cerebral blood flow in male mice (Figure 3A). Similarly, the results show that there is no effect of injection of saline vehicle (n = 12), 12 million CD4^+^ T cells (n = 11), or 8 million CD8^+^ T cells (n = 11) by intravenous transfer 24 h before MCAO on cerebral blood flow in female mice (Figure 3B).

### Adoptive transfer of activated CD4/CD8 T cells, 24 h before MCAO, does not increase infarct volume in splenectomized male WT mice

**Figure 4:**
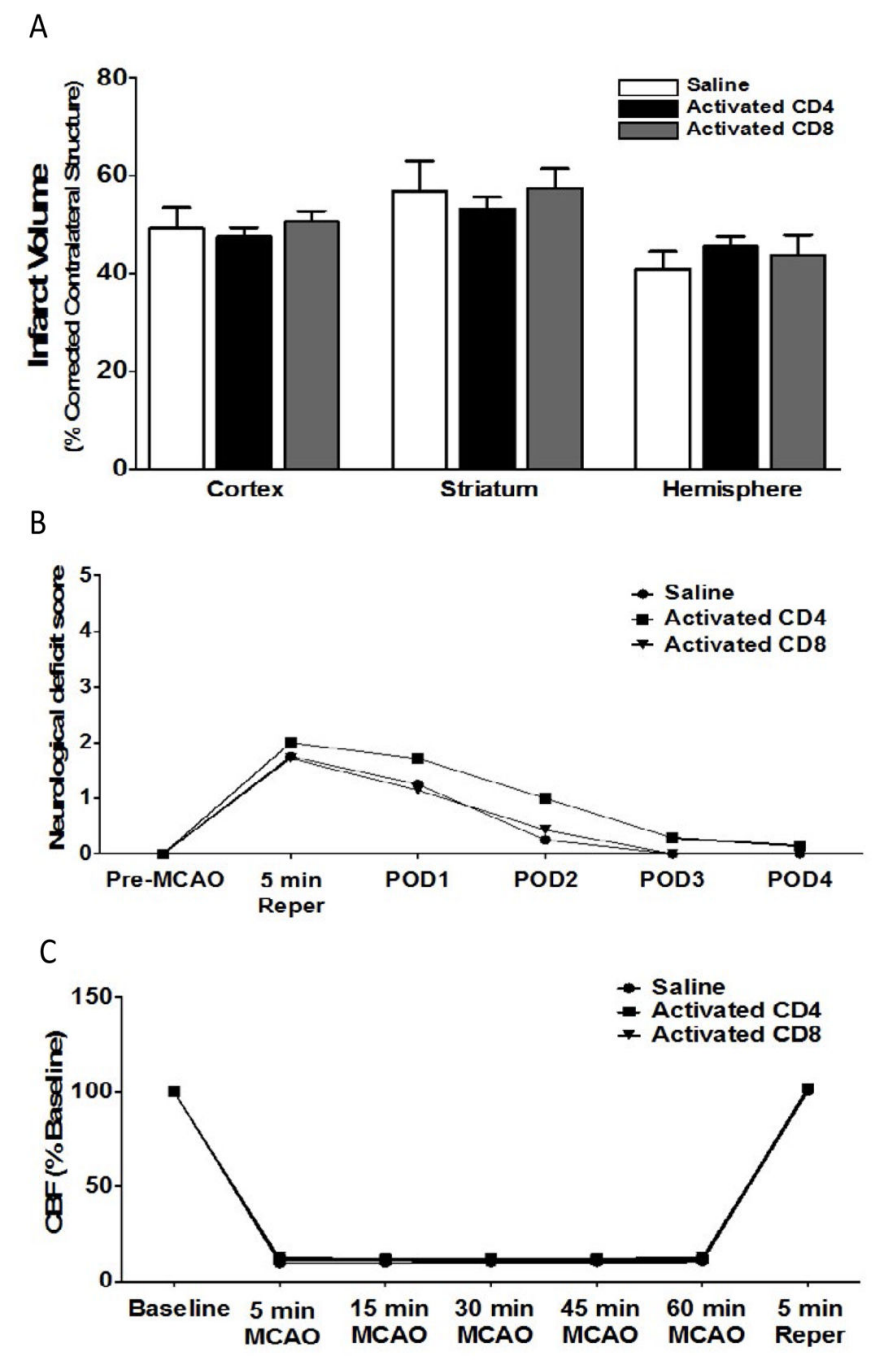
Adoptive transfer of activated CD4/CD8 T cells, 24 h before MCAO, does not increase infarct volume in splenectomized male WT mice Mice were injected with activated CD4^+^ or CD8^+^ T cells 24 hours prior to MCAO. Brains were harvested 96 h after MCAO. (A) Infarct volumes measured as percentage of corrected contralateral structure for male mice injected with saline vehicle (n=4), 12 million CD4^+^ T cells (n=7), or 8 million CD8^+^ T cells (n=7) by intravenous transfer 24 h before MCAO. (B) Neurological deficit score measured in male mice injected with saline vehicle (n=4), 12 million activated CD4^+^ T cells (n=7), or 8 million activated CD8^+^ T cells (n=7) by intravenous transfer 24 h before MCAO. (C) Cerebral blood flow measured in male mice injected with saline vehicle (n=4), 12 million activated CD4^+^ T cells (n=7), or 8 million activated CD8^+^ T cells (n=7) by intravenous transfer 24 h before MCAO. Values represent mean numbers (± SEM) of splenectomized mice per group analyzed using a one-way ANOVA. Functional outcomes for neurological deficit scores were analyzed by Mann Whitney Rank Sum test. Statistical significance was p<0.05.

Given the lack of effect of CD4 and CD8 adoptive transfer into male and female mice on ischemic injury (Figure 1), we then examined the effect of transferring activated CD4^+^ and CD8^+^ T cells on infarct volume in male mice. Donor mice were immunized with MOG, a brain antigen that is known to induce strong pathogenic T cell responses in rodents in vivo. Mice were injected with saline (n = 4), 12 million activated CD4^+^ T cells (n = 7), or 8 million activated CD8^+^ T cells (n = 7) 24 h prior to MCAO. Brains were harvested 96 h after MCAO. Figure 4A shows that there is no effect of intravenous transfer of activated CD4^+^ or CD8^+^ T cells on infarct volumes, measured as percentage of corrected contralateral structure. Values represent mean numbers (± SEM) of splenectomized mice per group analyzed using a one-way ANOVA. Figure 4B shows the neurological deficit scores measured in male mice injected with saline vehicle (n = 4), 12 million activated CD4^+^ T cells (n = 7), or 8 million activated CD8^+^ T cells (n = 7) by intravenous transfer 24 h before MCAO. Functional outcomes for neurological deficit scores were analyzed by Mann Whitney Rank Sum test. Statistical significance was *p*<0.05. Figure 4C shows cerebral blood flow measured in male mice injected with saline vehicle (n = 4), 12 million activated CD4^+^ T cells (n = 7), or 8 million activated CD8^+^ T cells (n = 7) by intravenous transfer 24 h before MCAO. Values represent mean numbers (± SEM) of splenectomized mice per group analyzed using a one-way ANOVA.

### Adoptive transfer of activated CD11b^+^ monocytes, 24 h before MCAO, does not increase infarct volume in splenectomized male and female WT mice

**Figure 5:**
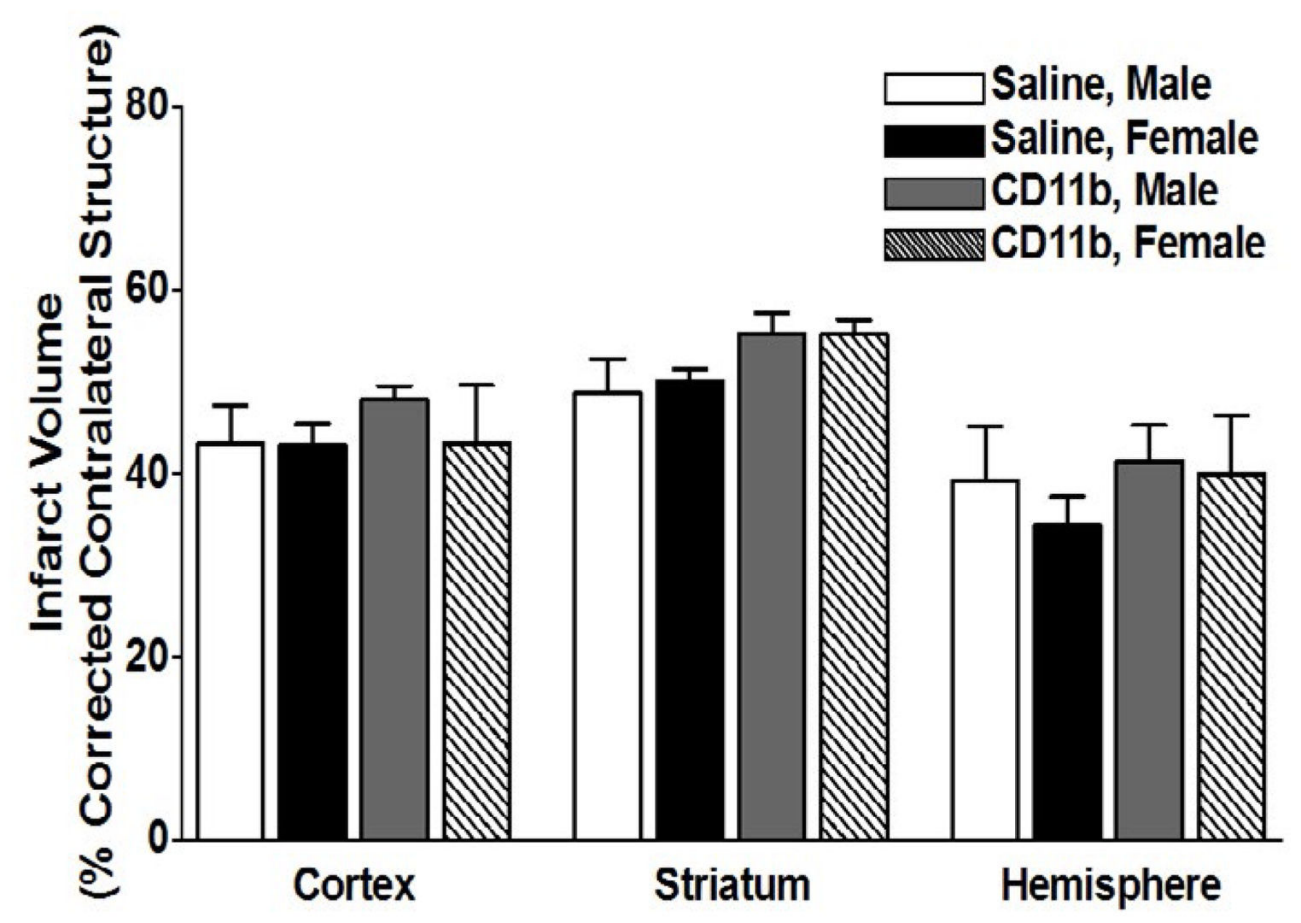
Adoptive transfer of activated CD11b^+^ monocytes, 24 h before MCAO, does not increase infarct volume in splenectomized male and female WT mice Male and female mice were subject to splenectomy two weeks prior to transient MCAO (60 min). Mice were injected with CD11b^+^ 24 hours prior to MCAO. Brains were harvested 96 h after MCAO. (A) Infarct volumes measured as percentage of corrected contralateral structure for male mice (n=3) and female mice (n=3) injected with saline vehicle, or male mice (n=5) and female mice (n=5) injected with 5 million CD11b^+^ cells. Values represent mean numbers (± SEM) of splenectomized mice per group analyzed using a one-way ANOVA.

We then examined the effect of adoptive transfer of activated CD11b^+^ monocytes on infarct volume in splenectomized male and female mice. Mice were injected with 5 million activated CD11b^+^ cells 24 hours prior to MCAO, and brains were harvested 96 h after MCAO. Figure 5 shows infarct volumes measured as percentage of corrected contralateral structure for male mice (n = 3) and female mice (n = 3) injected with saline vehicle, or male mice (n = 7) and female mice (n = 5) injected with 5 million activated CD11b^+^ cells. Values represent mean numbers (± SEM) of splenectomized mice per group analyzed using a one-way ANOVA.

### Effect of adoptive transfer of CD4^+^ and CD8^+^ cells on frequency of T cells in blood after MCAO in splenectomized male and female WT mice

**Figure 6:**
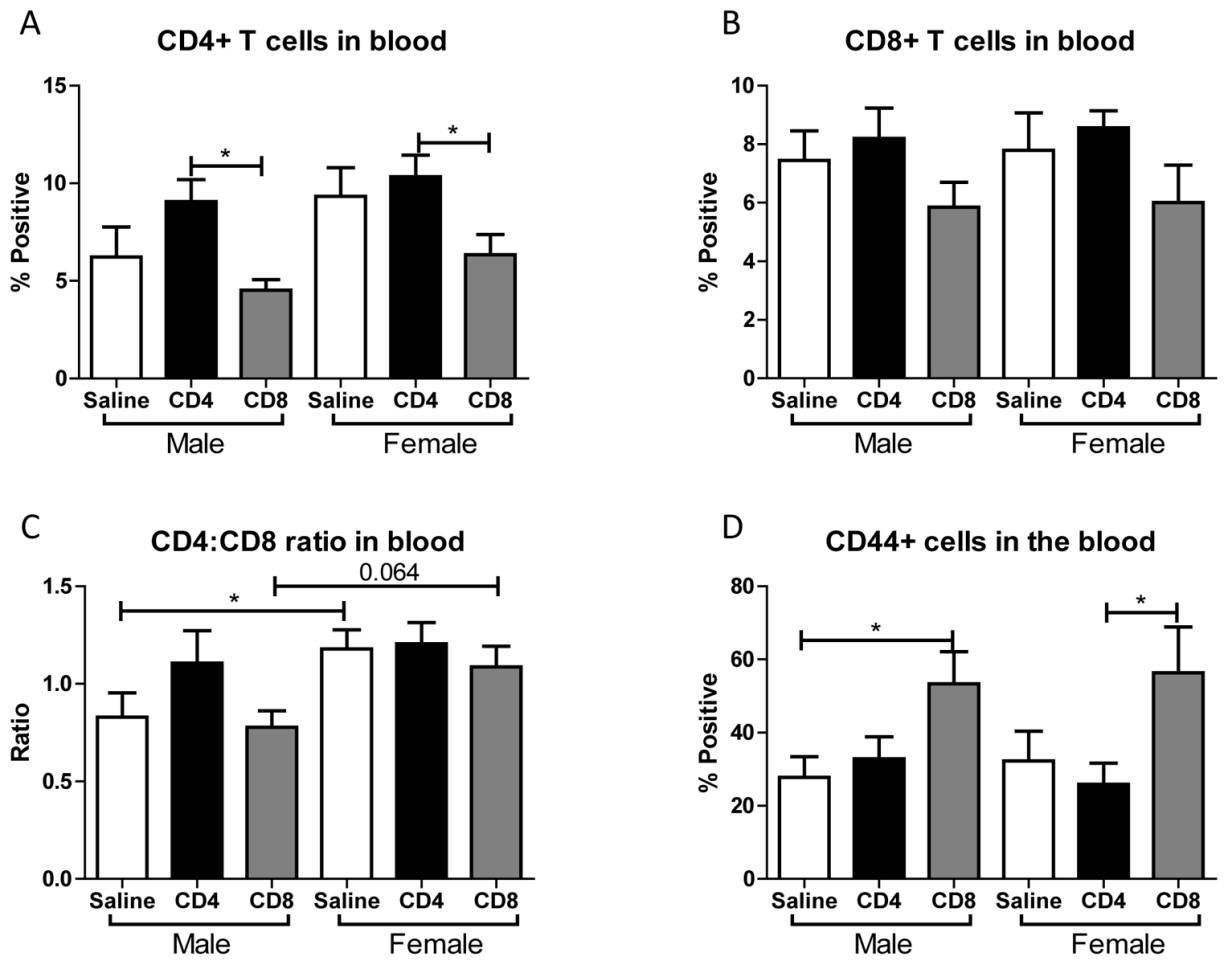
Effect of adoptive transfer of CD4/CD8 T cells on blood frequencies of CD4^+^, CD8^+^, and CD44^+^ in splenectomized male and female WT mice Blood was harvested 96 hours after MCAO from splenectomized mice and immunopheno-typed by flow cytometry. The frequencies of CD4+ and CD8+ T cells were determined (A, B). CD4:CD8 ratio was determined by dividing the frequency of CD4^+^ cells by the frequency of CD8^+^ cells (C). The frequency of cells expressing CD44 was also examined (D). Values represent mean numbers (± SEM) of the following groups: male saline n=6, male CD4 n=4, male CD8 n=4, female saline n=7, female CD4 n=8, female CD8 n=5. * indicates p<0.05 by t-test.

Given that adoptive transfer of CD4^+^ or CD8^+^ T cells to splenectomized male or female mice did not exacerbate infarct volume induced by focal ischemia (Figure 1), we examined the effect of MCAO on resident, non-GFP T cell subset frequencies in blood following adoptive transfer of unactivated CD4^+^ and CD8^+^ into male and female mice. Figure 6A shows that CD4^+^ transfer increases CD4^+^ T cells in the blood after MCAO, while CD8^+^ transfer decreases CD4^+^ T cells in the blood after MCAO. In contrast, Figure 6B shows that CD8^+^ T cells in the blood were largely unaffected after MCAO by transfer of either CD4^+^ or CD8^+^. Figure 6C shows that females exhibit an increased baseline CD4:CD8 ratio in the blood after MCAO (supported by Dotson, Wang *et. al.*), which was not significantly altered by CD8^+^ transfers and resulted in a loss of sex difference in CD4^+^ transfers. Figure 6D shows that transfer of CD8^+^ cells prior to MCAO increases the frequency of CD44^+^ (activated) cells in the blood.

### Effect of adoptive transfer of CD4^+^ and CD8^+^ on frequency of other immune subsets in blood after MCAO in splenectomized male and female WT mice

**Figure 7:**
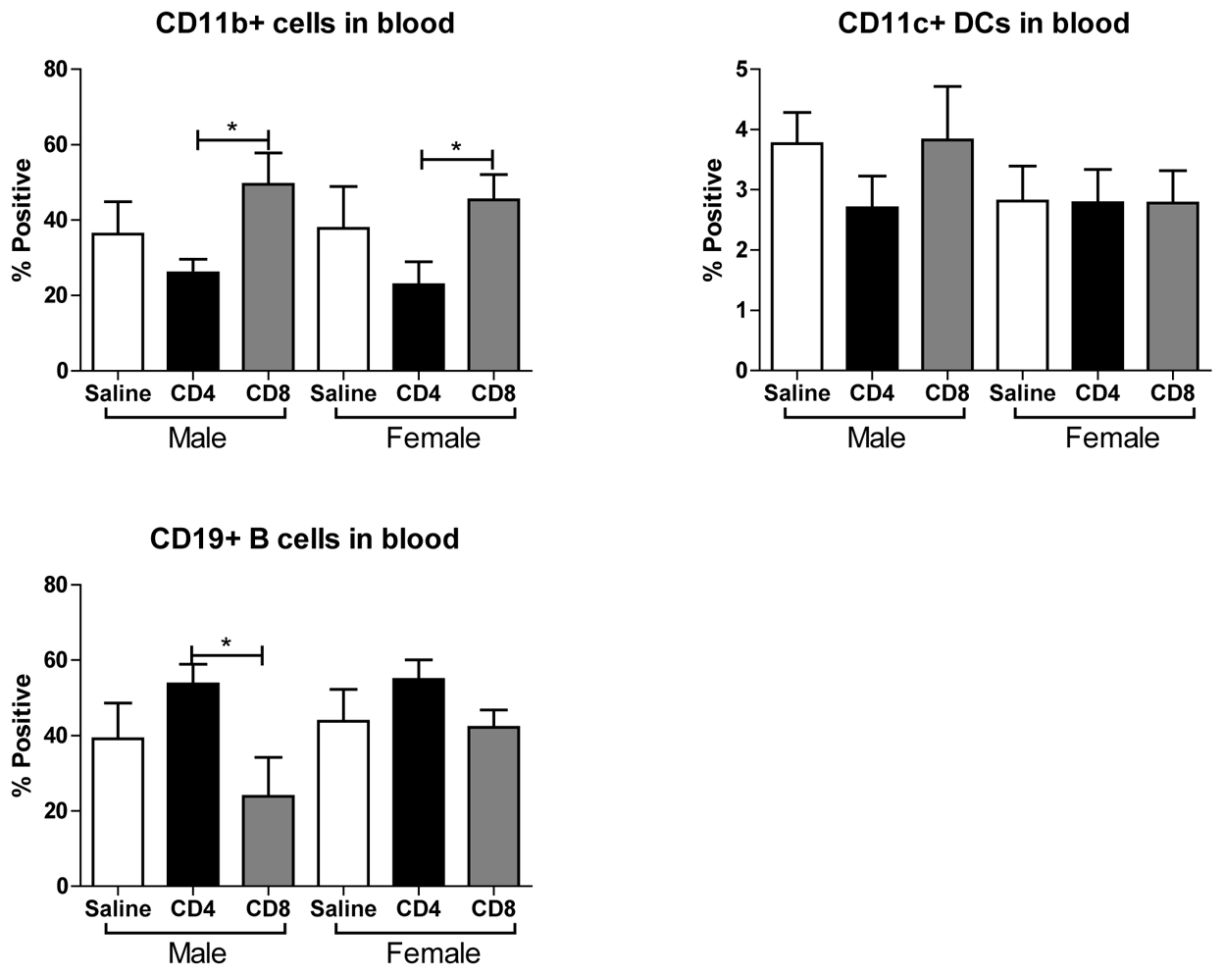
Effect of adoptive transfer of CD4^+^ and CD8^+^ on frequency of CD11b^+^, CD11c^+^ and CD19^+^ cells in blood after MCAO in splenectomized male and female WT mice MCAO from splenectomized mice and immunophenotyped by flow cytometry. The frequencies of CD11b^+^, CD11c^+^ and CD19^+^ cells were determined (A,B,C). Values represent mean numbers (± SEM) of the following groups: male saline n=6, male CD4 n=4, male CD8 n=4, female saline n=7, female CD4 n=8, female CD8 n=5. * indicates p<0.05 by t-test.

We further examined the effect of MCAO on CD11b^+^, CD11c^+^ and CD19^+^ cells in blood following adoptive transfer of unactivated CD4^+^ and CD8^+^ into male and female mice. Figure 7A shows that transfer of CD8^+^ cells prior to MCAO increases the frequency CD11b^+^ monocytes/ macrophages in the blood. In contrast, Figure 7B shows that transfer of either cell type does not significantly affect the frequency of positive CD11c^+^ dendritic cells in blood. However, Figure 7C shows that transfer of CD8^+^ cells prior to MCAO decreased the frequency of potentially protective CD19^+^ B cells in the blood in males, but this effect is less pronounced in females. These studies support that transfer of CD8^+^ T cells prior to MCAO leads to more potentially harmful immune cell subsets in the blood.

## Discussion

Males experience larger infarct volumes in response to experimental stroke than young females in both rats [11] and mice [12,13]. Many factors influence these differential outcomes to stroke, including infiltrating activated monocytes/microglia into the brain and T cell subsets in the periphery [13]. Splenic atrophy, a hallmark of post-stroke peripheral immune activation, is also significantly reduced in young female mice compared to male mice subjected to experimental stroke [12,13]. Our recent studies compared spleen intact to splenectomized male and female mice to determine the effects of the peripheral immune response sex differences to ischemia [8]. We found that infarct volume in young female mice is smaller compared to males, and in splenectomized males compared to spleen-intact males following experimental stroke, and that sex differences in infarct volume were eliminated by splenectomy. We further found that there is a significant difference in the ratio of CD4:CD8 in blood between spleen-intact males and females following MCAO, and that this difference correlated with CD4^+^ T cells in the periphery of the male mice. These findings were consistent with our previous studies, which showed increased CD4^+^ T cells in the spleens of male versus female mice following stroke [13]. Thus, we tested the effect of CD4^+^ and CD8^+^ transfer 24 hours before MCAO on stroke injury in splenectomized male and female mice. Given previous reports on the importance of T cells in stroke [14], we were surprised that adoptive transfer of either CD4^+^ or CD8^+^ before MCAO does not exacerbate stroke in male splenectomized mice (Figure 1A, B), or in female mice (Figure 1C, D). However, neurological deficit scores for male (Figure 2A) and female (Figure 2B) mice show that MCAO was successful, consistent with the decrease in cerebral blood flow measure before, during and after the MCAO in male (Figure 3A) and female (Figure 3B) mice. We then examined whether adoptive transfer of activated CD4^+^ and CD8^+^ cells was necessary to induce ischemic injury. MOG is a brain structural component thought to leak into the periphery following brain injury. MOG stimulated, and therefore brain antigen specific, splenocytes significantly exacerbate infarct volume in MCAO SCID mice compared to naïve splenocytes [15]. Again, our studies did not yield expected results as they revealed that adoptive transfer of activated CD4^+^ and CD8^+^ cells 24 hours before MCAO also did not worsen stroke in male mice (Figure 4A) although neurological deficit scores (Figure 4B) and cerebral blood flow measures before, during and after the MCAO (Figure 4B) show that the MCAO was successfully performed. Based on these outcomes, we did not test the effect of adoptive transfer of activated CD4^+^ and CD8^+^ cells in female mice.

Experimental stroke leads to an increase in circulating CD11b^+^ monocytes [3] which can secrete inflammatory cytokines such as IL- 1β, IL-6, TNFα that maintain the inflammatory response and result in tissue-specific monocyte homing. Our previous studies also revealed that there is a significant increase in circulating CD11b+ monocytes in spleen-intact male mice when compared to splenectomized male mice following stroke, a difference that was not seen in female mice. Thus, we examined whether adoptive transfer of activated CD11b^+^ monocytes/ macrophages 24 hour before MCAO had any effect on infarct volume, and found, much like our T cell results, that there was no difference in injury outcome in males or female splenectomized mice (Figure 5).

Established research indicates a pathogenic role for T cells and macrophages in stroke and a lack of splenocytes leads to improved stroke outcome. It is, therefore, logical that reconstitution of splenocyte subsets into splenectomized mice prior to MCAO should yield an increase in infarct volume. However, previous successful adoptive transfer studies in males were done on T and B cell deficient mice [14]. Splenectomized mice have been shown to exhibit leukocytosis [16,17], possibly interfering with the transferred cells. Another possibility is that not enough cells were transferred to directly affect infarct outcome. Previous reports transfer 50–100 million splenocytes, whereas we were only transferring a spleen equivalent number of individual cell subsets, an amount up to ten times lower. Additionally, it is probable that the cell subsets act together in the spleen and in total splenocyte transfers and that by isolating cell subsets, the ability of the individual subsets to efficiently activate one another was lost.

Although the transferred cell subsets were not sufficient alone to worsen infarct, we did observe immunomodulation of the resident blood immune cells in the periphery after MCAO when the mice received unactivated T cells. Throughout these studies we examined the outcome of adoptive transfer of CD4^+^ and CD8^+^ T cells on their expression in blood. We found that adoptive transfer of CD4^+^ but not CD8^+^ T cells led to a significant increase in blood levels of CD4^+^ in male and female mice (Figure 6A). However, adoptive transfer of CD8^+^ did trend towards decreased levels of blood CD4^+^ and CD8^+^ T cells in male and female mice (Figures 6A and 6B). The ratio of CD4^+^ to CD8^+^ T cells in blood was significantly higher in splenectomized females that received no cells than males (Figure 6C), as previously reported [8]. A similar trend in peripheral CD4:CD8 ratio is exhibited with adoptive transfer of CD8^+^, but not CD4^+^, T cells (Figure 6C). Additionally, adoptive transfer of CD8^+^ but not CD4^+^ T cells increased the frequency of CD44^+^ cells in the blood of male and female mice (Figure 6D). Adoptive transfer of CD8^+^ but not CD4^+^ T cells increased the frequency of CD11b^+^ cells in the blood of male and female mice (Figure 7A). Yet adoptive transfer of CD4^+^ T cells appeared to cause an increase in CD19^+^ B cells in the blood, and a decrease of CD19^+^ B cells in mice receiving CD8^+^ T cells (Figure 7C) although this difference was not significant in female mice. Together, the data reveal that adoptive transfer of CD8^+^ T cells 24 hours prior to MCAO led to immune cell activation and an increase in potentially pathogenic macrophages/ monocytes in the periphery while transfer of CD4^+^ T cells yields more potentially protective B cells. Additionally, a only small percent (<1% of total cells) of GFP^+^ cells were detected in the blood and a slightly larger percent (3–4% of total cells) of GFP^+^ cells were detected in the lymph node (data not shown) demonstrating that major shifts in resident immune cell frequencies were the result of a relatively small transferred population.

This study demonstrates that adoptive transfer of a spleen equivalent number of CD4^+^ or CD8^+^ T cells prior to MCAO does not lead to harmful effects on infarct injury in male or female splenectomized mice, nor does the adoptive transfer of activated CD4^+^ or CD8^+^ T cells or CD11b^+^ monocytes/macrophages prior to MCAO alter infarct outcomes in male or female splenectomized mice. It is probable that more cells or all immune subsets are needed to impact infarct volume. However, this study demonstrates that adoptive transfer of CD8^+^ T cells prior to MCAO leads to more potentially harmful immune cell subsets in the blood.

## Highlights

- We reconstituted individual immune subsets in splenectomized mice before MCAO
- Unactivated CD4 or CD8 T cells did not affect infarct volume or neurological deficit
- Activated CD4, CD8 or CD11b cells did not affect infarct volume or neurological deficit
- Lack of effect could be a result of splenectomy, insufficient cells or separated subsets
- CD8 T cells lead to more activated and possibly harmful immune cell subsets in the blood

## Abbreviations

CNS: Central nervous system
MCAO: Middle cerebral artery occlusion
WT: wild-type
GFP: Green fluorescent protein
CD: Cluster of differentiation
MOG: Myelin oligodendrocyte glycoprotein
PBS: Phosphate-buffered saline
FACS: Fluorescence activated cell sorter
